# Effectiveness and user experience of a virtual reality intervention in a cohort of patients with chronic musculoskeletal pain syndromes

**DOI:** 10.1371/journal.pdig.0000788

**Published:** 2025-03-31

**Authors:** Tiffany Prétat, Pedro Ming Azevedo, Chris Lovejoy, Thomas Hügle

**Affiliations:** Department of Rheumatology, University Hospital Lausanne (CHUV) and University of Lausanne, Lausanne, Switzerland; Iran University of Medical Sciences, IRAN, ISLAMIC REPUBLIC OF

## Abstract

Chronic musculoskeletal pain (CMP) syndromes, including fibromyalgia, present diverse physical and psychological symptoms often resistant to pharmacological treatment. To retrospectively evaluate the effectiveness and user experience of Virtual Reality (VR) in reducing pain and anxiety in CMP patients and identify predictors of positive response. Data from 91 CMP patients in a 2-week interdisciplinary pain program were analyzed (78% met fibromyalgia criteria). Pain and anxiety were assessed using Numerical Rating Scales (NRS 0-10) before and after VR. Follow-up interviews were conducted after one month. An unsupervised machine learning model explored response patterns. VR led to a moderate but significant short-term reduction in anxiety and pain (median NRS −1.0, p < 0.001). A reduction of ≥3 NRS points occurred in 25% (anxiety) and 14% (pain). High baseline anxiety (NRS ≥ 7) correlated with greater pain reduction (median −2.0, p = 0.01). After one month, half of the patients reported sustained benefits. Catastrophizing and benzodiazepine use were linked to improved anxiety outcomes. Machine learning identified a most responsive cluster, characterized by patients with nociplastic pain, alexithymia, and anxiety. VR provided moderate short- and mid-term benefits for anxiety and pain in CMP patients, particularly in those with nociplastic pain and high baseline anxiety.

## Author summary

Chronic musculoskeletal pain (CMP), including conditions like fibromyalgia, is often difficult to manage with traditional medications alone. This study explores the potential of Virtual Reality (VR) as a tool to alleviate pain and anxiety in patients with CMP. We analyzed data from 91 patients who participated in a two-week interdisciplinary pain program and used VR as part of their treatment. During the program, comprehensive psychological and rheumatological assessments, as well as sleep analysis, were performed. Our findings indicate that VR provided moderate short-term relief, with nearly half of the participants reporting reduced anxiety and pain. Interestingly, patients with high levels of anxiety before the VR session experienced the most significant improvements. A follow-up survey conducted one month later showed that over half of the participants continued to perceive benefits from VR, particularly in reducing anxiety. A machine learning model helped identify specific patient characteristics that predicted a stronger response to VR, including those with nociplastic pain and psychological factors such as anxiety and alexithymia. While VR was well received by most participants, some experienced discomfort due to positioning or visual effects. This study highlights VR’s potential as a non-pharmacological option for managing chronic pain and anxiety, especially for patients with significant psychological distress. Future research should explore long-term effects and further personalize VR therapy for optimal outcomes.

## Introduction

Chronic pain represents the first most common reason internationally for years lived with disability and for healthcare consultations in the US [1,2]. Unlike in acute pain, classical analgesic pharmacological treatments have only a modest effect in chronic musculoskeletal pain (CMP) syndromes such as fibromyalgia, e.g., due to nociplasticity and overlapping anxiety or depression [3–5]. Nociplastic pain indicate that the central nervous system is involved in pain perception and chronicity, often associated with central hypersensitization. EULAR recommendations encourage a patient-centered, individualized approach for patients with fibromyalgia, where the use of medications is considered carefully, with a strong preference for low-dose and non-opioid treatment such as amitriptyline, duloxetine or gabapentin [3]. These guidelines highlight the importance of a balanced approach that prioritizes non-pharmacological therapies while carefully incorporating drug treatments when required for symptom relief. Furthermore, side effects and dependence on drugs such as opioids may complicate the disease course and may promote chronicity [6]. There is evidence for a plethora of efficacious non-pharmacological interventions for CMP, such as physical exercise, cognitive behavioral therapy, or mindfulness [7–9]. Multimodal treatment programs have shown notably positive effects in fibromyalgia [10].

Virtual reality (VR) is increasingly used in the non-pharmacological treatment of chronic pain, including musculoskeletal pain [11–12]. Three concepts emerge about this technology: the capacity to immerse the user through multi-sensory inputs, the capacity to create the perception of presence inside the virtual environment and finally the possibility of interacting with the virtual environment [13]. Beyond analgesia, VR has been shown to have a positive effect on general anxiety disorders, specific phobias, or post-traumatic stress disorder (PTSD) and the effect seems to be comparable to in vivo exposure therapy [14]. Patients with high levels of stress but no psychiatric disorders also benefit from VR and it can be used as a tool to decrease kinesiophobia [15,16]. There are contradictory results regarding functional outcomes such as range of motion [17–19].

In this study, we applied a VR intervention to a CMP cohort who underwent an extensive interdisciplinary pain assessment, as described elsewhere [20]. We assessed short- and midterm VR effectiveness and identified patients’ characteristics predictive of positive response. The high prevalence of psychiatric diagnoses such as depression, anxiety and PTSD in patients with CMP is important for stratifying the response of patients to VR. To our knowledge this is the first study using a broad panel of psychosomatic variables to study this topic and to use an unsupervised machine learning model for clustering analyses.

## Methods

### Ethics statement

The study was approved by the local ethical commission (CER-VD Project-ID 2021-00853) and written informed consent was obtained by all patients.

Patients 91 adult patients with CMP undergoing VR interventions during a two-week rheumatology-led multimodal pain program at the University Hospital Lausanne, Switzerland were included. Inclusion criteria for the program were persistent musculoskeletal pain in one or more body regions, refractory to different types of pain medication, physiotherapy and interventional approaches such as infiltration or neuro-ablation.

Patient characteristics are shown in Table 1. <5% of the patients had prior experience with VR of another type. Exclusion criteria for the program were the existence of cognitive, physical, and severe active psychiatric comorbidities preventing patients from understanding and adhering to procedures. Patients needed to be at least 18 years old and speak French fluently.

**Table 1:** Patients’ characteristics.

**Demography**							
Female	Age (mean, SD)		Foreign origin	BMI (median, range)	In couple		Parenthood
70/91 (76.9%)	47.7 (+/−10)		45 (49.5%)	26.2 [16.5; 50]	52/91 (57.1%)		59/91 (64.8%)
**Clinical history**							
Hyperlaxity	Fibromyalgia criteria fulfilled. (ACR 2010 or FiRST)			Waddell signs (Median, range)	Immune-mediated disease		Pain since childhood or adolescence
28/82 (34.1%)	32/41 (78.0%)			1, [0; 5]	9/90 (10%)		42/58 (72.4%)
**Type of pain**							
Nociceptive	Nociplastic		Functional	Neuropathic peripheral	Inflammatory (active arthritis)		
63/91 (69.2%)	77/91 (84.6%)		63/91 (69.2%)	16/90 (17.8%)	3/90 (3.3%)		
**Psychiatric comorbidities**							
Psychiatric diagnosis^*^	Depression		Anxiety	PTSD	EPCACE		Alexithymia
72/91 (79.1%)	53/91 (58.2%)		28/91 (30.8%)	12/91 (13.2%)	16/91 (17.6%)		41/91 (45.1%)
**Sleep evaluation**							
Effectiveness				**Fragmentation index**			
Median, range		85.4%, [21.2%; 93.4%]		Median, range		25.8, [0.18; 85.4]	
Decreased <85%		38/79 (48.1%)		Index >20		60/79 (75.9%)	

*Alexithymia is not included in psychiatric diagnosis. PTSD: Post traumatic stress disorder, EPCACE: Enduring Personality Change After Catastrophic Experience.

### VR intervention

The VR software and hardware used consisted of an immersive environment with a 360° vision field and audio-hypnosis (Healthymind, France) with a Pico G2 4 K VR headset (Pico Interactive Inc., San Francisco, California, USA), as described elsewhere [21]. Patients could choose between six environments (forest, Japanese garden, snowy or sunny mountain, beach, or scuba-diving). Patients completed standardized breathing exercises following visual clues for respiratory cycles. All patients underwent two sessions of 20 minutes each, one week apart. Patients were usually lying down on an examination couch with pillows to minimize pain due to positioning. An example of the virtual environment (beach) is seen in S1 Fig.

### VR effectiveness and user experience

Numerical Rating Scale (NRS, 1-10) was assessed for pain and anxiety before and directly after the first session (short-term effect). Patients also evaluated the VR experience directly after the session as positive, neutral, or negative. One month after the program (mid-term effect) a semi-structured telephone interview was performed assessing their experience of VR in general, in relation to other therapeutic interventions and their effect on pain and anxiety.

### Clinical variables

Demographic, clinical, and psychiatric variables are listed in S1 Table. The Pain Catastrophizing Scale (PCS) was completed the day before VR. Psychiatric diagnosis by a specialist included depression, GAD (generalized anxiety disorder), PTSD (Post traumatic stress disorder) or EPCACE (Enduring Personality Change After Catastrophic Experience) and other psychiatric comorbidities (bipolar or personality disorders, alexithymia). Analgetic and psychotropic drugs were also included. Patients completed various questionnaires, including the Brief Pain Inventory (BPI). The Fibromyalgia Rapid Screening Tool (FiRST) [22] and the American College of Rheumatology (ACR) 2010 criteria [23] were used to diagnose fibromyalgia. Sleep fragmentation was measured using actigraphy (MotionWatch 8), with a Sleep Fragmentation Index (SFI) threshold set at 20 [24]. Additional clinical and demographic variables were used for machine learning model (S2 Table), including menopausal status and laboratory values (C-reactive Protein, sedimentation rate).

### Statistical analysis

The primary endpoints were defined as reduction in pain and anxiety, both assessed using NRS before and after the VR intervention. Secondary endpoints were User Experience of VR evaluated directly after the VR session as positive, neutral, or negative. A semi-structured telephone interview was performed one month after the program to assess their experience of VR in general, in relation to other therapeutic interventions, and its effect on pain and anxiety. Mid-term impact on pain and anxiety was assessed via a follow-up survey conducted one month after the intervention.

To study pain or anxiety reduction, patients without pain (n = 2) or anxiety (n = 23) at baseline of the program were excluded. We selected statistical tests by taking into account the nature of variables (e.g., dependent or independent, metric or categorical) and distribution of data. Distributions were non-parametric. Significance of delta NRS was analyzed through Wilcoxon tests to compare two dependent categorical samples to a metric dependent variable with non-parametric distribution). Delta NRS were compared with Mann-Whitney U (MW-U) tests between patients with baseline high pain/anxiety level (≥7 on NRS) and low pain/anxiety level (≤6). Categories of patients with high vs. low NRS levels were compared using Chi-squared tests. The strength of correlations for significant results was tested using Spearman´s correlation. Categorical demographic, clinical and psychiatric variables were compared with MW-U tests, while numeric variables were analyzed via Spearman’s correlation. Association directions were assessed using box plots and scatter plots. All analyses were controlled for age, sex and alexithymia.

### Unsupervised machine learning

A machine learning clustering model was pre-trained in 123 patients of the same multimodal cohort previously described [20]. Clusters were generated using the features described in S2 Table). We applied hierarchical agglomerative clustering using the Ward variance minimization algorithm with Euclidean pairwise distances. With this method, the new entry d(u, v) is computed as follows:


$$d\left(u,v\right)=\sqrt{\frac{\left|v\right|+\left|s\right|}{T}d{\left(v,s\right)}^{2}+\frac{\left|v\right|+\left|t\right|}{T}d{\left(v,t\right)}^{2}-\frac{\left|v\right|}{T}d{\left(s,t\right)}^{2}}$$

where u is the newly joined cluster consisting of clusters s and t, v is an unused cluster in the forest, T = |v| + |s| + |t|, and |*| is the cardinality of its argument. The analysis was performed using the SciPy cluster library. Principal components analysis (PCA) was applied to clusters using the scikit-learn library, and the two components explaining the most variance were visualized on a 2-dimensional scatterplot. The optimal number of clusters was determined a posteriori through visual examination after cluster visualization (including the dendrogram and the two-dimensional PCA plot) using matplotlib and seaborn libraries. The response to VR was compared between clusters using a one-way analysis of variance (ANOVA). A Bonferroni adjustment was applied to a significance level of 0.05 for p-values, to identify variables with statistically significant variation between clusters. Analyses were conducted using Stata/MP 13.0. Clustering was performed using Python 3.10. Variables were grouped into continuous variables and categorical variables. Variables were compared using appropriate tests: two-sample proportion tests for binary outcomes and chi-square tests for categorical outcomes. Correlations were calculated using point biserial correlations for correlations between binary and continuous variables. Accordingly, clusters were compared using a one-way analysis of variance for continuous variables, as listed in S2 Table. A Bonferroni adjustment was applied to a signiﬁcance level of 0.05 for P values to identify variables with statistically signiﬁcant variation among clusters. Two experienced rheumatologists performed the clinical interpretation of clusters.

## Results

### Effects of VR on anxiety and pain

**Fig 1:**
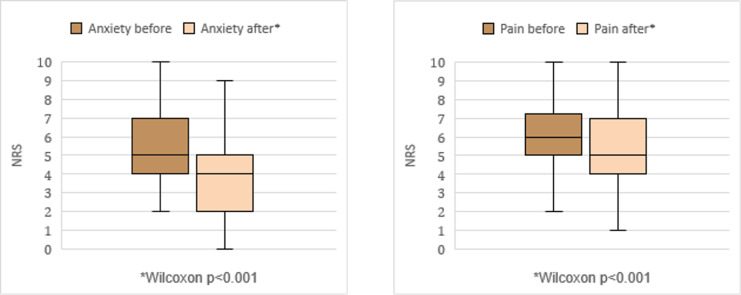
Pain and anxiety levels before and after VR. Pain and anxiety were measured on a NRS scale 0–0 (max) in patients with baseline pain >1.

Directly after the intervention, 49.4% of all patients reported a decrease in anxiety, 43.5% reported no change, and 7.1% stated that it increased. Pain decreased for 51.2% of patients, remained the same for 33.7%, and increased for 15.1%. Both pain and anxiety decreased in 31.8% of patients, while neither changed for 17.6%. Among patients with a baseline NRS greater than 1, 67.7% reported decreased anxiety, 25.8% reported no change, and 6.5% reported an increase. The mean and median delta scores in anxiety (mean −1.0, median 0, p < 0.001, Fig 1) and pain (mean −0.9, median −0.75, p < 0.001) decreased significantly (Fig 1, S3 Table). Two participants reported nausea but continued the experience.

### Predictive factors of a positive response to VR

Patients with high baseline anxiety ≥7 benefited more from VR than those with lower levels of anxiety in terms of both anxiety and pain reduction (Table 2). Conversely, patients with high baseline pain (NRS ≥ 7) experienced similar benefits from VR as those with lower pain levels. The distribution of delta anxiety scores is shown in the supplement S2 Fig. Catastrophizing and the use of benzodiazepines were significantly associated with improved outcome of VR on anxiety, but not on pain (Table 3). The association of Benzodiazepines/Z-drugs and the Pain Catastrophizing Scale (PCS) with delta anxiety is shown in S3 Fig.

**Table 2:** Response to VR depending on anxiety and pain at baseline (NRS >1).

**Anxiety groups**									
	**N, %**	**Δ Anxiety** (median range, 95% CI*)		MW-U*	**Spearman**		**Δ Pain** (median range, 95% CI)	**MW-U**	**Spearman**
**High ≥7**	21/62 (33.9%)	−3, [−9; 0], [−4; −1]		**p = 0.002**	p = 0.001, r = −0.40		−2, [−6; 1], [−3; 0]	**p = 0.010**	p = 0.009 r = −0.33
**Low ≤6**	41/62 (66.1%)	−1, [−4; 3], [−1; 0]					0, [−6; 2], [−1; 0]		
**Pain groups**									
	**N, %**	**Δ Anxiety** (median range, 95% CI*)		**MW-U**			**Δ Pain** (median range, 95% CI*)	**MW-U**	
**High ≥7**	37/84 (44.0%)	−1, [−9; 7], [−3; 0]		p=0.239			0, [−6; 1], [−1; 0]	p = 0.945	
**Low ≤6**	47/84 (56.0%)	0, [−6.5; 2], [−1; 0]					−1, [−5; 2], [−1; 0]		
**Age (mean, SD), sex (N,%) and alexithymia (N,%) repartition**									
**Group**	**High anxiety**		**Low anxiety**			**High pain**		**Low pain**	
**Age**	49.0 +/− 8.6		47.1 +/− 10.0			46.8 +/− 10.4		48.6 +/− 10.3	
Sex (F*)	16/21 (76.2%)		32/41 (78.1%)			27/37 (73.0%)		39/47 (83.0%)	
**Alexithymia**	10/21 (47.6%)		17/41 (41.5%)			19/37 (51.4%)		15/47 (31.9%)	

* MW-U = Mann-Whitney U test, F = female sex.

**Table 3:** Univariate analysis of epidemiological and clinical characteristics.

	Δ anxiety		Δ pain	
Sub-group (N)	All patients (N = 85)	Baseline NRS > 1, (N = 62)	All patients (N = 86)	Baseline NRS > 1, (N = 84)
Mann-Whitney U tests	p-value	p-value	p-value	p-value
Sex	0.439	0.206	0.306	0.213
In couple	0.704	0.521	0.708	0.480
Children	0.765	0.498	0.871	0.931
Foreign origin	0.919	0.937	0.604	0.584
Nociceptive back pain	0.188	0.605	0.484	0.395
Nociceptive peripheral pain	0.439	0.528	0.271	0.137
Nociplastic pain	0.171	0.453	0.460	0.355
Neuropathic peripheral pain	0.279	0.750	0.829	0.962
Radiculopathy	0.324	0.591	0.746	0.630
Functional pain syndromes	0.685	0.917	0.394	0.325
Pain since childhood or adolescence^±^	0.079	0.440	0.494	0.953
FM criteria fulfilled.	**0.054**	0.279	0.148	0.099
Depression^**^	0.638	0.933	0.629	0.643
GAD^**^	0.804	0.513	0.554	0.629
PTSD or EPCACE^**^	0.196	0.219	0.632	0.743
Alexithymia^**^	0.996	1.000	0.801	0.866
Other psychiatric conditions^**^	0.412	0.821	0.938	0.839
Hyperlaxity^§^	0.538	0.798	0.502	0.204
Fragmented sleep^¥^	0.480	0.309	0.898	0.967
Efficient sleep^¥^	0.098	**0.055**	0.117	0.217
Paracetamol/ Metamizol	0.219	0.183	0.971	0.722
NSAIDs	0.803	0.835	0.561	0.804
Opiates	0.543	0.721	0.966	0.751
Benzodiazepines & Z-drugs	0.022*	0.093	0.066	0.099
**Spearman’s correlation**				
Age	0.678	0.996	0.357	0.343
BMI	0.838	0.853	0.679	0.816
Waddell score	0.127	**0.054**	0.956	0.863
Pain Catastrophizing Scale (PCS)	0.002*	0.073	0.128	0.237

§ Hyperlaxity according to medical anamnesis, including but not solely according to Beighton’s score.

± Mostly recurrent “growing pain”, sprains and musculoskeletal injuries, abdominal pain, headache, back pain and invalidating dysmenorrhea.

* In both cases, the association is positive.

** Diagnose according to a psychiatric evaluation and not questionnaires. GAD = generalized anxiety disorder, PTSD = post-traumatic stress disorder, EPCACE = enduring personality change after a catastrophic event. Other psychiatric conditions included bipolar disorder, personality disorders and alexithymia.

¥ Fragmented sleep & efficient sleep according to actigraphy.

There was a trend toward a correlation between response to VR for anxiety and fulfilled fibromyalgia criteria, sleep efficiency and Waddell scores. No statistically significant differences were found in age (p = 0.419) or sex (p = 0.267) between the high and low pain groups. However, the proportion of patients with alexithymia was higher in the high pain group than in any other group. This did not achieve significance when compared with the low pain group (p = 0.072).

### Overall VR user experience

**Fig 2:**
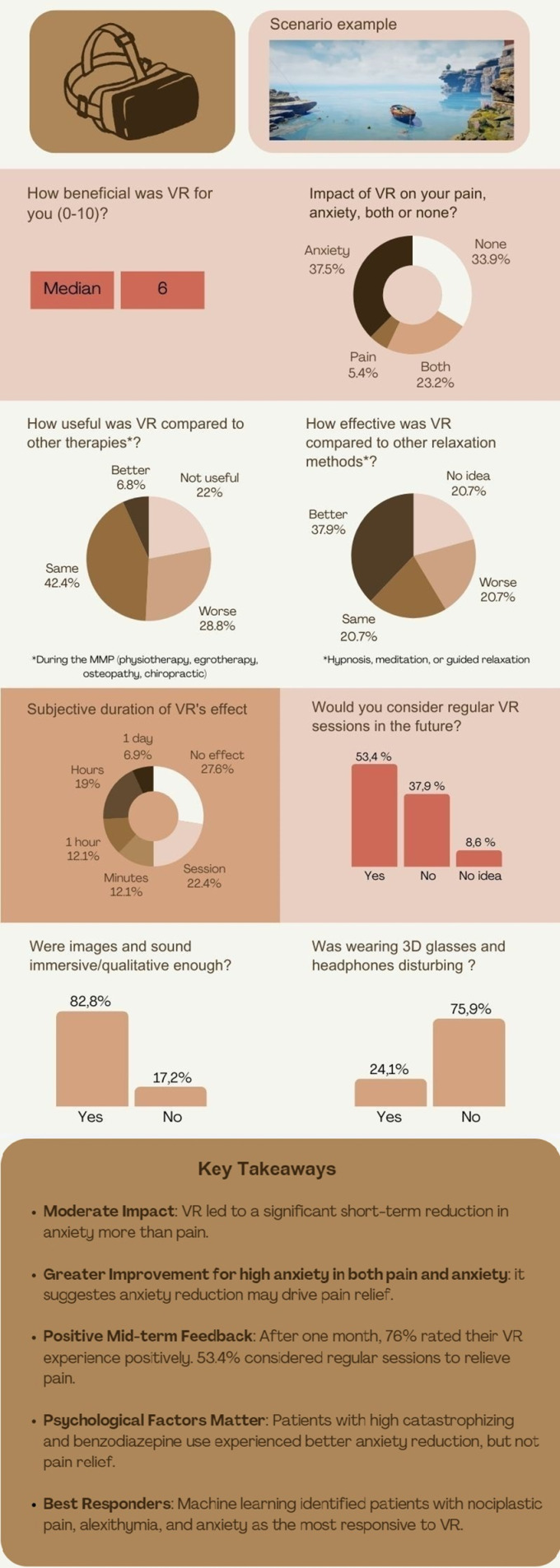
Infographic presenting the results of the telephone survey on VR user experience.

The overall VR experience was rated positive by 76% of participants, neutral by 14%, and negative by 8%. One month later, more than half of the patients reported that VR had a positive impact on their anxiety, pain, or both, with a particularly strong benefit for anxiety. Forty-two percent of patients found the VR intervention as beneficial as other treatments received during the multimodal program, such as physiotherapy, occupational therapy, chiropractic care, or osteopathy. 58% considered VR to be as effective or even more effective than traditional relaxation techniques such as hypnosis, meditation, or guided relaxation. However, a quarter of the participants felt its effects lasted only a few hours to a day. Positive feedback about VR mentioned its immersive qualities that allowed users to momentarily escape from their worries and pain, the audio-visual support that enhanced focus more effectively than hypnosis, the high-quality imagery, and the engaging nature akin to video games. Negative experiences were attributed to pain from prolonged improper posture, recent pain flare-ups, or discomfort due to room temperature. Some found the game-like elements juvenile, and others were inadvertently disturbed by their imagination, such as interpreting shadows as something sinister. Criticisms also included dissatisfaction with the unrealistic aspects and quality of the visuals. The 3D glasses and headphones posed practical challenges for individuals who wear prescription glasses. Two participants reported nausea (cybersickness) but could continue the experience. Six participants (7%) reported anxiety directly caused by the VR visuals and sounds. Pain worsening after VR was attributed to positioning. A summary of the outcomes from telephone interviews conducted one month after the intervention is shown in the form of an infographic (Fig 2).

### Clustering analysis

**Fig 3:**
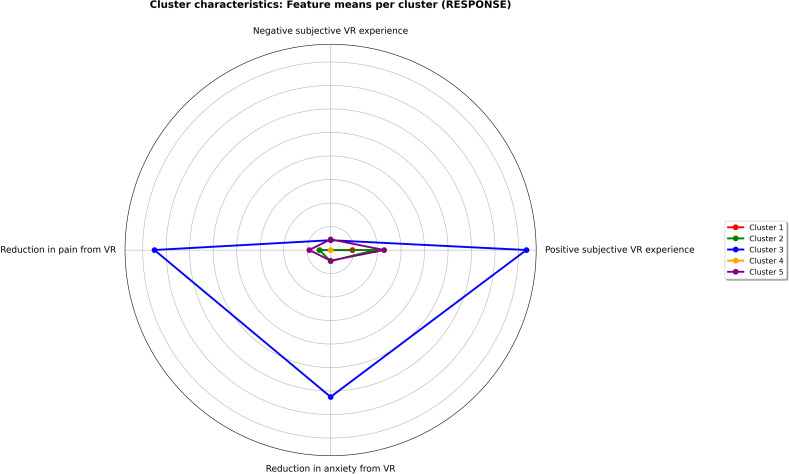
Radar plots for the five clusters. Each axis of the radar plot represents a different variable. Clusters are represented by a distinct color within the same chart. Reduction of pain and anxiety from VR (Virtual Reality) referred to short term response directly after the intervention. Positive or negative subjective VR experience was rated one month after the intervention.

An unsupervised machine learning model, pre-trained on 123 patients from the multimodal program, identified five clusters representing distinct musculoskeletal pain types among participants. Out of these, 63 patients underwent two cycles of VR treatment. The distribution of patients across the clusters was as follows: cluster one had 9 out of 44 patients, cluster two had 9 out of 21, cluster three included 23 out of 24, cluster four comprised 7 out of 13, and cluster five contained 15 out of 21 patients. Fig 3 shows a radar plot with a blue line highlighting cluster three, which showed a superior response to VR in terms of short-term reduction of pain and anxiety, along with a subjectively positive intervention experience reported after one month. Cluster three was characterized by patients with nociplastic pain, alexithymia, and pre-existing anxiety or depression disorders.

## Discussion

This study reports a moderate short-term effect of VR on pain in patients with CMP. The impact on anxiety surpassed that on pain reduction and appeared to be more enduring. Patients with higher baseline anxiety levels experienced a more pronounced effect on pain, suggesting that the mechanism through which VR alleviates pain is at least partly mediated by anxiety reduction. Previous studies have highlighted VR’s positive effect on anxiety [17,18]. In contrast to previous VR studies in fibromyalgia, we provide more detailed information on patient subtypes and a one month follow-up including user experience and effectiveness.

The sustained positive user experiences related to anxiety, reported by the majority of patients one month after the intervention, were particularly noteworthy. Half of the patients considered VR to be as effective as other therapeutic interventions included in the multimodal treatment, such as physiotherapy, occupational therapy, or chiropractic therapy. The global response of VR in our cohort (S3 Table) was comparable with pain reduction achieved by VR in other studies [25]. The calculated effect size (Cohen’s *d*) for the reductions in pain and anxiety in the study is 0.375 for pain reduction and 0.4 for anxiety reduction. This is comparable to other non-pharmacological treatments for fibromyalgia such as physical exercise programs or cognitive behavioral therapy (CBT) [8].

Given the limitations of the NRS in capturing the multifaceted effects of pain or anxiety, the assessment of user experience is becoming increasingly important in non-pharmaceutical interventions for chronic pain. Additionally, almost half of the patients in this cohort were diagnosed with alexithymia, which may hinder their ability to accurately report emotions on an NRS scale and thus express the effects of VR through user experience rather than numerical ratings. No significant safety signals of VR intervention were observed. Minor issues reported led to improvements in our VR intervention after the study, such as adapting posture, or room temperature or chair positioning. 15.1% of the participants reported increased pain after the intervention. This could be partially due to subjective variation of reported PROs such as pain on an NRS scale, stress-related or due to wrong positioning.

Although VR has been studied in fibromyalgia, this is the first report of its use in a multimodal chronic pain program. The observed positive effects on pain and anxiety suggest that VR could be a valuable and scalable component of such multimodal pain management programs for CMP patients, particularly those with elevated anxiety levels. Some smaller studies have explored VR’s effect on anxiety in chronic pain syndromes, such as chronic back pain, noting trends towards significant anxiety reduction compared to controls [26,27]. Our analysis not only aligns with these findings but also indicates that patients using benzodiazepines or those prone to catastrophizing respond better to VR in terms of anxiety reduction, though not pain relief. We observed a tendency for a greater VR response to anxiety in patients with preserved sleep, positive Waddell signs, and fulfilled fibromyalgia criteria. Potentially, patients with better sleep have improved emotional regulation and cognitive function, allowing them to engage more effectively with VR interventions. While the positive effect of VR on sleep has been demonstrated in various studies [28], more research is needed to understand how sleep quality impacts VR’s effectiveness in fibromyalgia patients. The association of VR effectiveness with positive Waddell signs and the fulfillment of fibromyalgia criteria may suggest that VR interacts with central sensitization. However, we did not identify any variables associated with a positive VR response for general pain or specific pain types, such as nociplastic pain. Although patients with alexithymia were more prevalent in the high-pain group, the relationship between alexithymia and VR’s effect on pain did not reach statistical significance, indicating that further research is needed to clarify this relationship. Psychiatric comorbidities in form of depression, GAD, PTSD did not show a significant association with VR’s impact on pain or anxiety, although psychological factors were noted as important overall. In fact, having an anxiety disorder may not necessarily mean being anxious before VR, and therefore, it may not correlate with a better anxiety response. This is also why studying the effect of VR not only in a predefined setting could provide more meaningful information.

Further statistical analyses such as multivariate regression analysis or ANOVA could describe relationships between those variables in more detail, ideally in a larger dataset. By adjusting for confounding variables, this analysis could provide more precise estimates of which factors significantly predict VR effectiveness.

The main limitations of this study include a small sample size, lack of a control group, short intervention duration, psychological biases in the patient population, and the challenges of isolating VR’s effects within a multimodal treatment program. Several variables are subjective or interpreted by the examiner such as nociplastic pain. While the user experience is reported, a more qualitative analysis of patient feedback could provide richer insights into the perceived benefits and drawbacks of the VR intervention. Self-reported measures remain a key limitation when interpreting the results. Future studies should incorporate more objective endpoints, such as physiological markers of anxiety or pain, to complement the self-reported data.

Nevertheless, 53% of participants in the telephonic survey after one month believed they would benefit from regular VR sessions, with 25% reporting symptom relief ranging from a few hours to an entire day. It should be noted here that other treatment modalities from the multimodal program may have influenced the VR user experience. We omitted metrics for the unsupervised machine learning model such as F1, precision or recall due to the low patient number per cluster.

The study confirms VR’s heterogenic effect in CMP management, with varying levels of interactivity and immersion potentially influencing analgesia and anxiolysis effectiveness. Studies suggest that higher interactivity and immersion levels could enhance VR’s effectiveness, as active participation in the virtual environment may distract from pain sensations [29]. The increasing accessibility of VR pain and relaxation programs, alongside technological advancements, positions VR as a scalable option for chronic pain patients [30]. The integration of VR with technologies like biofeedback or vagal stimulation could amplify VR’s pain-relieving effects [31].

By applying machine learning to cluster patients in our multimodal pain program, we predicted VR response, revealing clinically relevant phenotypes with mental health features as significant discriminators [32].. A specific cluster, characterized by younger patients with significant psychiatric comorbidities, including alexithymia, responded best to VR. The importance of alexithymia has recently been investigated in a machine learning model based on an alexithymia scale where it accurately distinguished patients from fibromyalgia controls [33]. This underscores the potential role of VR in addressing alexithymia, but also factors like solitude, as previously noted in fibromyalgia studies.

Our results should be confirmed in larger patient cohorts, and further cross-validation analyses should be performed to reduce the risk of overfitting. Another important limitation is the consistency and standardization of clinical variables. While we carefully selected and harmonized key variables used in our clustering model, differences in data collection methods across studies may pose challenges for replication. Establishing standardized definitions for key variables, such as pain type classification and psychiatric comorbidities, will be crucial for future multicenter investigations. Incorporating objective physiological markers (e.g., heart rate variability, cortisol levels) in future studies could provide additional insights into VR’s mechanisms of action. Validation of the model in external datasets is an important next step to confirm the effectiveness of VR in different clusters. Confirming the higher effectiveness of VR interventions in cluster three-like patients through a randomized controlled trial would support the development of personalized treatment approaches for fibromyalgia

Overall, this study demonstrates that this VR intervention has differential impacts across various patient subgroups, with the most significant effects observed in those with high baseline anxiety, nociplastic pain, and psychological comorbidities such as alexithymia. While VR shows great potential in reducing anxiety and, to a lesser extent, pain, its effectiveness is modulated by factors such as sleep quality, psychological profile, and the nature of the pain experienced. These findings underscore the importance of a personalized approach when integrating VR into multimodal pain management programs, particularly for patients with significant psychological distress and central sensitization syndromes like fibromyalgia.

## Supporting information

S1 Fig Figure: Example of virtual environment used (beach). (DOCX)

S2 Fig Repartition for delta anxiety (N = 85) and pain (N = 86). (DOCX)

S3 Fig Direction of association for Benzodiazepines/Z-drugs combined and Pain catastrophizing scale (PCS) with delta anxiety in all patients. (DOCX)

S1 Table Clinical variables used for the univariate analysis. (DOCX)

S2 Table Variables used for the machine learning model. (DOCX)

S3 Table Global response to VR treatment. (DOCX)
