# Supplementary figures and images for: Effectiveness and user experience of a virtual reality intervention in a cohort of patients with chronic musculoskeletal pain syndromes
Source: PLOS Digit Health. 2025 Mar 31;4(3):e0000788. doi: 10.1371/journal.pdig.0000788 (PMC11957290; doi:10.1371/journal.pdig.0000788)

S1 Figure: Example of virtual environment used (beach)


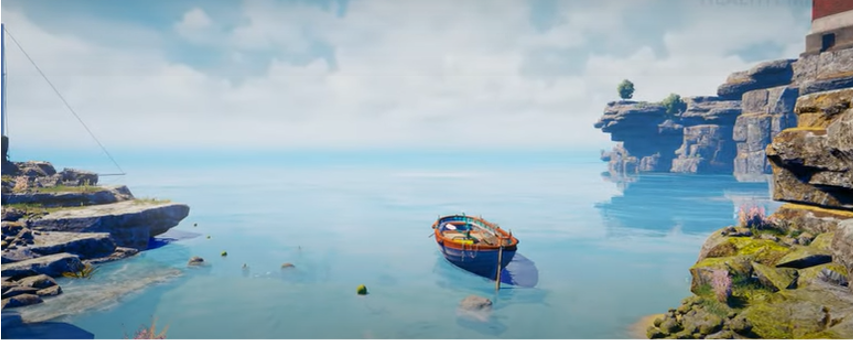

Supplement: S1 Fig — (DOCX) [file pdig.0000788.s001.docx]

Supplement S2 Figure: Repartition for delta anxiety (N=85) and pain (N=86).


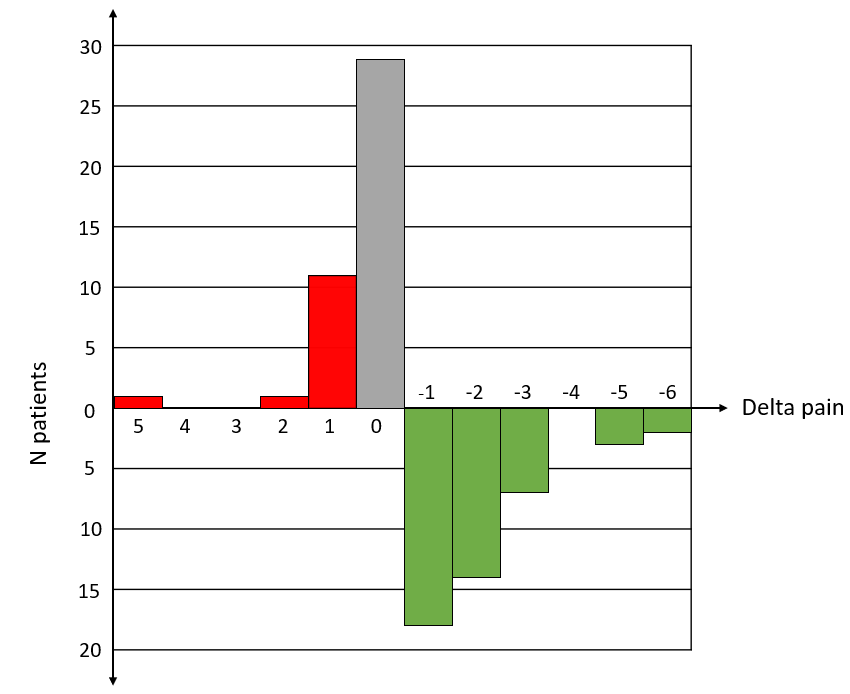

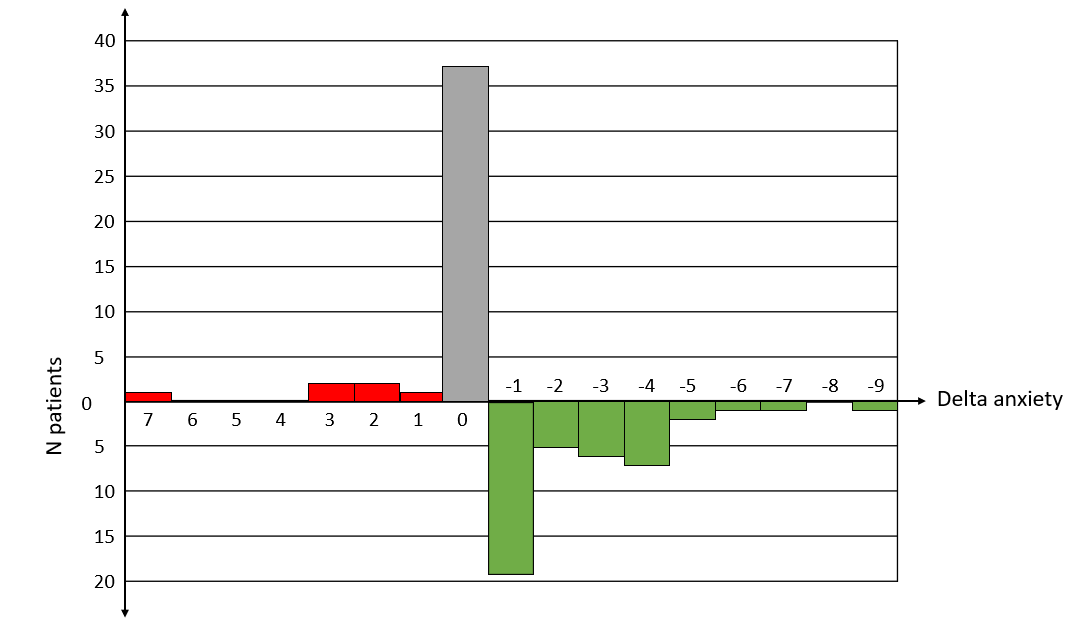

Supplement: S2 Fig — (DOCX) [file pdig.0000788.s002.docx]
