# Supplementary material for: Effectiveness and user experience of a virtual reality intervention in a cohort of patients with chronic musculoskeletal pain syndromes
Source: PLOS Digit Health. 2025 Mar 31;4(3):e0000788. doi: 10.1371/journal.pdig.0000788 (PMC11957290; doi:10.1371/journal.pdig.0000788)

Supplement S3 Figure: Direction of association for Benzodiazepines/Z-drugs (0= no, 1= yes) combined and Pain catastrophizing scale (PCS) with delta anxiety in all patients.


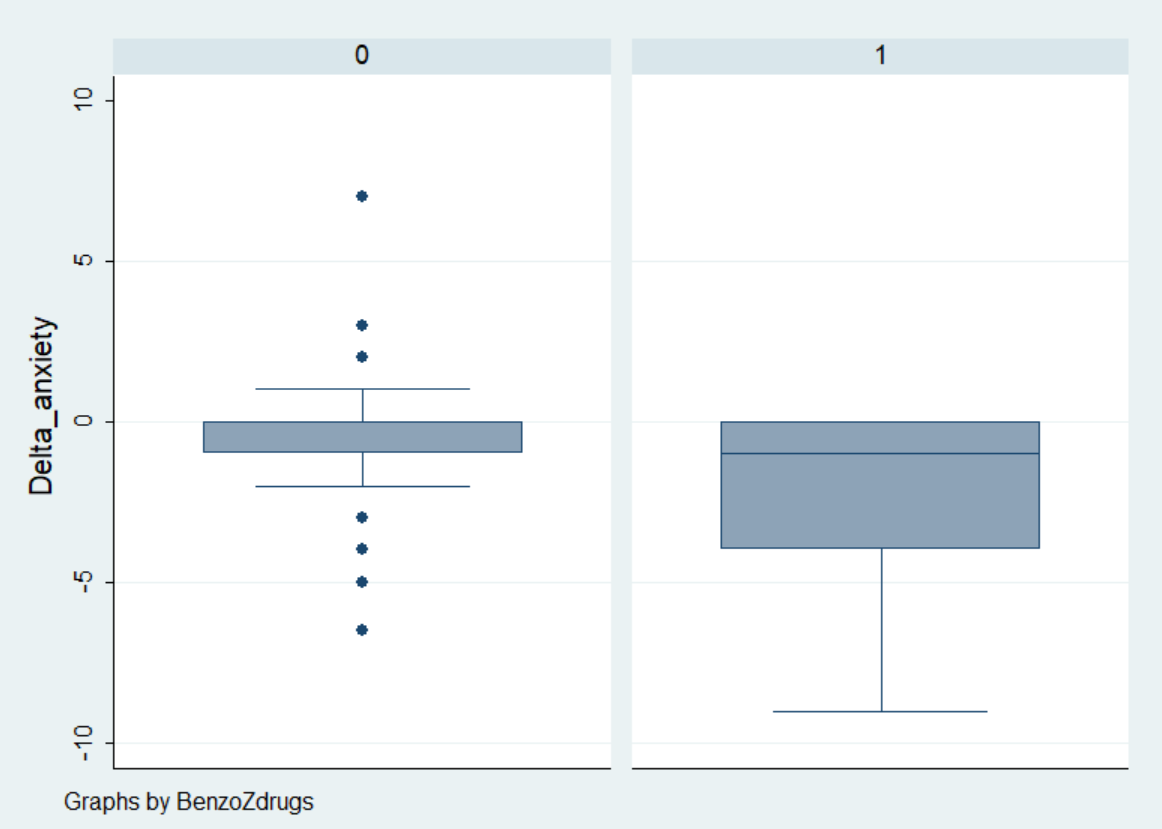


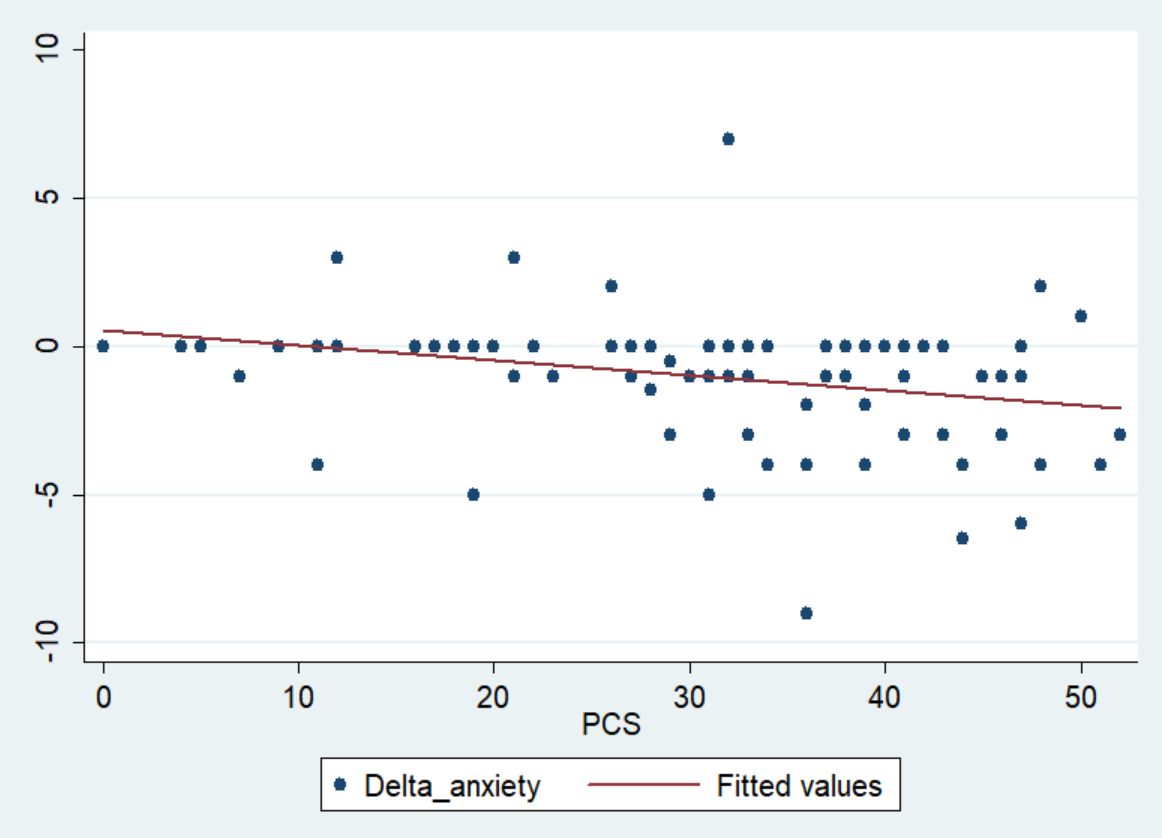

Supplement: S3 Fig — (DOCX) [file pdig.0000788.s003.docx]
