# Supplementary material for: Effectiveness and user experience of a virtual reality intervention in a cohort of patients with chronic musculoskeletal pain syndromes
Source: PLOS Digit Health. 2025 Mar 31;4(3):e0000788. doi: 10.1371/journal.pdig.0000788 (PMC11957290; doi:10.1371/journal.pdig.0000788)
Supplement: S1 Table — (DOCX) [file pdig.0000788.s004.docx]

Supplement S1 table: Clinical variables used for the univariate analysis

| **Phenotypic Domain** | **Clinical Variables** |
| --- | --- |
| Demographics | Age, sex, relationship status (single or in couple), presence of children, foreign origin |
| Pain | Nociceptive back/peripheral pain, nociplastic pain, peripheral neuropathic pain and radiculopathy. Functional pain syndromes. number of Waddell signs. Presence of chronic pain since childhood or adolescence. |
| Comorbidities | Fulfillment of FM criteria (ACR 2010 and FiRST) Depression, GAD, PTSD or EPCACE, other psychiatric conditions (any of: bipolar disorder, personality disorder, alexithymia) |
| Other characteristics | BMI (continuous, no categories), hyperlaxity, sleep efficacy, sleep fragmentation* |
| Medications | Paracetamol/ Metamizole, NSAIDs, Opiates (weak and strong), benzodiazepines & Z-drugs |
| *Sleep efficacy and sleep fragmentation index are determined by actigraphy during one week follow-up. Sleep is considered not efficient if <85% of the night is spent sleeping. Sleep is considered fragmented if the sleep fragmentation index >20.  FM=fibromyalgia, ACR=American College of Rheumatology, FiRST=Fibromyalgia Rapid Score Screening Test, GAD=generalized anxiety disorder, PTSD=post-traumatic stress disorder, EPCACE=enduring personality change after catastrophic experience, BMI=Body Mass Index. Neuropathic, nociceptive or nociplastic pain character were defined by the clinician. | |
