# Supplementary material for: Effectiveness and user experience of a virtual reality intervention in a cohort of patients with chronic musculoskeletal pain syndromes
Source: PLOS Digit Health. 2025 Mar 31;4(3):e0000788. doi: 10.1371/journal.pdig.0000788 (PMC11957290; doi:10.1371/journal.pdig.0000788)
Supplement: S2 Table — (DOCX) [file pdig.0000788.s005.docx]

Supplement S2 Table: Variables used for the machine learning model

| **Phenotypic Domain** | **Clinical Variables** |
| --- | --- |
| Demographics | Age, sex, relationship status (single vs in couple), presence of children, disability insurance status (demanded vs received vs refused), time taken off work. |
| Pain | Back pain, peripheral pain (any of: peripheral neuropathy, significant peripheric arthrosis, enthesopathy, bursitis, tendinopathy, shoulder pain), neuropathic pain (any of: radiculopathy, peripheral neuropathy), nociplastic pain, nociceptive pain, chronic pain since childhood or adolescence |
| Comorbidities | Immune-mediated rheumatic disease, airway comorbidities (any of: OSA, COPD, asthma), metabolic disease (any of: diabetes, gout), depression or anxiety, PTSD, EPCACE, other psychiatric conditions (any of: bipolar disorder, personality disorder, alexithymia) |
| Other  characteristics | BMI* (<18.5 vs 18.5-25 vs 25-30 vs >30), menopausal status, hyperlaxity, disturbed sleep* |
| Medications | Opiates (weak vs strong), Antidepressant treatment (tricyclics vs mirtazapine vs dual antidepressant therapy vs. trazodone vs sympathomimetics), neuroleptics, benzodiazepines, Z-drugs, NSAIDs, Prednisolone, Biologics (bDMARDs*) |
| **Defined as a fragmentation index of greater than 20 or sleep efficiency less than 85%, as measured using a MotionWatch 8. **As described by ICD-10 code F62.0  BMI=Body Mass Index, OSA=obstructive sleep apnea; COPD=chronic obstructive pulmonary disease; PTSD=post-traumatic stress disorder; EPCACE=enduring personality change after catastrophic experience, NSAIDs=non-steroidal anti-inflammatory drugs bDMARDs=biologic disease modifying anti-rheumatic drugs | |
