# Supplementary material for: Effectiveness and user experience of a virtual reality intervention in a cohort of patients with chronic musculoskeletal pain syndromes
Source: PLOS Digit Health. 2025 Mar 31;4(3):e0000788. doi: 10.1371/journal.pdig.0000788 (PMC11957290; doi:10.1371/journal.pdig.0000788)
Supplement: S3 Table — (DOCX) [file pdig.0000788.s006.docx]

Supplement S3 Table: Global response to VR treatment.

|  | **All patients** | **NRS > 1** | **Wilcoxon** |
| --- | --- | --- | --- |
| **Δ** **Anxiety** | | | |
| N data available, % | 85/91 (93.4%) | 62/91 (68.1%) | p<0.001 |
| Median, range | 0, [-9; 7] | -1, [-9; 3] |  |
| **Δ** **Pain** | | | |
| N data available, % | 86/91 (94.5%) | 84/91 (92.3%) | p<0.001 |
| Median, range | -0.75, [-6; 5] | -1, [-6; 2] |  |
